# Public School Children

**Published:** 1861-10

**Authors:** 


### PUBLIC SCHOOL CHILDREN.

In the name of humanity, this article is addressed to the thoughtful and affectionate consideration of those who have children at the public schools, of which New-York City may well be proud, because they are conducted generally with a system, a vigor, a thoroughness, and an efficiency, which merit our admiration, to say nothing of the gratitude due from this whole community to the company of teachers, whose literary ability and conscientious efforts to discharge fully the responsible duties of their station, have made these schools what they are. For the unmitigated barbarity and most consummate stupidity of keeping children from eight years old and upward, six mortal hours at a time, on hard benches, breathing a contaminated atmosphere, with only a few minutes' interval, now and then, with the addition of lessons to be learned at home, which we personally know, require from two to three hours more, on the part of those not particularly bright, the teachers are not responsible. But the Board of Education are. And any one of them who passively permits the continuation of the enormity another day, ought to be incontinently ejected from the Board, neck and heels. Such a man has not the sympathy, consideration, and intelligence, necessary to the management of a drove of Barnum's monkeys. He would kill the hippopotamus in a week. Old Adams's California giant bear would wither to a skeleton; every member of the "Happy Family," and every inhabitant of the multitudinous and beautiful aquaria, (all to be seen for only twenty-five cents!) would dwindle down to a bag of bones. We would not be surprised to learn that the keeper who killed off the two Labrador whales, was a public school commissioner. The law ought to be instantaneous and imperative, that no pupil under the junior class, should be allowed to take a book home, or to study one single moment out of the insufferable six hours. Until New-York can rise to the intelligence of the Boston Board, some plan like the following should be adopted in order to prevent the children from falling into habitual constipation, which lays the foundation for brain-fever; or the slower, but life-long calamity of a residence in some lunatic asylum; or, the brain, stimulated into diseased action, loses its power, and the unfortunate child, however bright and promising at first, soon reaches the acme of its capabilities, beyond which it can not go, and falls into jejune mediocrity.

Constipation, Dyspepsia, and Brain-Fever are the three great dangers of children at school. When a child gets to pass a day or two without an action of the bowels, it becomes at once exposed to every disease that is abroad; if there is any prevalent sickness whatever, a constipated child is sure to suffer from it; while it takes cold from the slightest of all causes, and thus becomes tinder for diphtheria, scarlet fever, and putrid sore-throat. Hence, breakfast should be taken early, long enough before half-past eight o'clock, to allow them to have abundant time to go to the privy; and to promote this, they should be required to repair to the family-room or parlor, from the breakfast-table; because, by the mind being composed, the call of nature is more certainly noticed. It is a criminal neglect, not to clearly explain to each child, the inevitable ill results and danger to life, which attend going over twenty-four hours, without an evacuation of the bowels.

A good many give their children money to buy cakes, candies, nuts, and other trash, to be eaten as lunch at the twelve o'clock recess. It is positively certain, that in every case such children will become dyspeptic in a few weeks, or otherwise disabled from attending school. The very best, and an abundant lunch, is one slice of light bread, made of what is called Graham or unbolted flour, mixed with molasses enough to make it slightly sweet. This will appease hunger, for it is very nutritious, will promote a free condition of the bowels, and yet allow the child to have a moderate but good appetite for dinner at three and a half clock, after which, nothing should be eaten but a couple of oranges. But as too indulgent parents may not be easily brought to this, a piece of cold bread and butter, or an equivalent of stirabout or cracked wheat, with a little molasses, is amply sufficient. The importunities of the little Olivers for "more," should be firmly and kindly resisted; then, instead of seeing the children come to the breakfast-table with a listless, weary, unrefreshed look, only nibbling at a bit of bread, or sipping a little water, they will be bright, cheerful, and able to eat almost any thing placed before them—provided they have had the utmost abundance of sleep; for if this is not allowed a child, not one in ten will see thirty years; and even those, from the day of marriage and on, will be years of annoying if not miserable invalidism. To ascertain what is sufficient sleep without mistake, and as some naturally require more sleep than others, there is only one safe plan of procedure, and that is as infallible as nature herself. Make the child go to bed at an earlier and earlier hour, until he wakes up of himself, about six o'clock; until nature wakes him up; which she will never fail to do as soon as enough sleep has been taken to repair the muscular and mental wear and tear of the preceding day.

If a child is put to bed at nine o'clock in the evening, and does not wake at six, after a week's trial, then try eight or seven, and when the proper hours are ascertained, adhere to them pretty rigidly; do not make any inflexible rule. If, from unavoidable irregularity, a child has to be waked up, do not trust a servant to do it. The parent should do it mildly, gently, pleasantly, encouragingly. Servants are very apt to do it with a shock, or scowl, or scold, or other exhibition of impatience, and the day of the child begins with a feeling of irritation which is very apt to color the whole of that day's conduct.

Two rules as to eating are of incalculable importance. If a child is under ten years of age, every particle of food should be cut up by one of the parents, in pieces almost as small as a pea, with a sharp knife; or if the servant does it, it should be brought to the parent for inspection, before it is placed before the child, and nothing should be eaten between meals but a piece of dry bread, or an orange, or ripe fruit, about midway between. Children over ten should not be allowed to eat any thing between meals except on some special occasion. Nothing so soon and so certainly wears out the stomach of a child as eating something every two or three hours.

By the time the public-school scholar gets through with dinner, it is about four o'clock. Let it be rigidly understood that they must keep on their feet out of doors, if not raining or otherwise inclement, until it is dark enough to come in, and then let them be busied in plays or games, or some household occupation which requires them to be actively employed on their feet until tea; after which let them have some amusement until retiring; always timing things so that they shall be ready to get into bed within five minutes of the regular hour; arranging all in such a way that not a single moment shall be employed in study, or reading, or sewing by gas, or candle, or other artificial light. Whatever lessons can not be learned between rising in the morning and the time for starting to school, let them remain unlearned. If, on application to the teachers, the out-of-school task can not be modified, then insist that the child be put in a lower class. It is infinitely better that a child shall remain a year longer at school, or to have no schooling at all, (excepting always a religious training,) and grow up with good health, than by incessant and painful stimulations have the brain over-strained with the inevitable result of a constitution blighted in its budding, to be a burden or a torture to life's latest hour.

Finally, parents, as you can never tell that any night shall not be the last on earth, however well the child may seem on retiring, and that it shall not wake up to a brain-fever, or dreaded croup, or the more fearful diphtheria, or putrid sore-throat, be persuaded to make a habitual and systematic arrangment by which each child shall retire to its little bed with a feeling of affectionate lovingness toward you; that no harsh word, or look, or inconsiderate act of yours shall ruffle its little heart, and cause it to turn its face to the wall against you. Your indifferent, stereotyped, matter-of-course kiss is a cruel hypocrisy. The little creatures perceive it by an instinct, and they lie down with an undefined unsatisfaction. If you do not feel a kiss, do not commit the atrocity of a mere form, but go and pray God to give you a better heart.

